# The physiology of invasive plants in low-resource environments

**DOI:** 10.1093/conphys/cot026

**Published:** 2013-11-05

**Authors:** Jennifer L. Funk

**Affiliations:** School of Earth and Environmental Sciences, Chapman University, Orange, CA 92866, USA

**Keywords:** Invasion biology, leaf economics spectrum, resource acquisition, resource conservation, restoration ecology

## Abstract

While invasive plant species primarily occur in disturbed, high-resource environments, many species have invaded ecosystems characterized by low nutrient, water, and light availability. Species adapted to low-resource systems often display traits associated with resource conservation, such as slow growth, high tissue longevity, and resource-use efficiency. This contrasts with our general understanding of invasive species physiology derived primarily from studies in high-resource environments. These studies suggest that invasive species succeed through high resource acquisition. This review examines physiological and morphological traits of native and invasive species in low-resource environments. Existing data support the idea that species invading low-resource environments possess traits associated with resource acquisition, resource conservation or both. Disturbance and climate change are affecting resource availability in many ecosystems, and understanding physiological differences between native and invasive species may suggest ways to restore invaded ecosystems.

## Abstract (precis)

This review examines physiological and morphological traits of native and invasive species occurring in environments characterized by low nutrient, water and light availability. Species invading low-resource environments possess traits associated with resource acquisition, resource conservation, or both acquisition and conservation.

## Introduction

**Figure 1.**
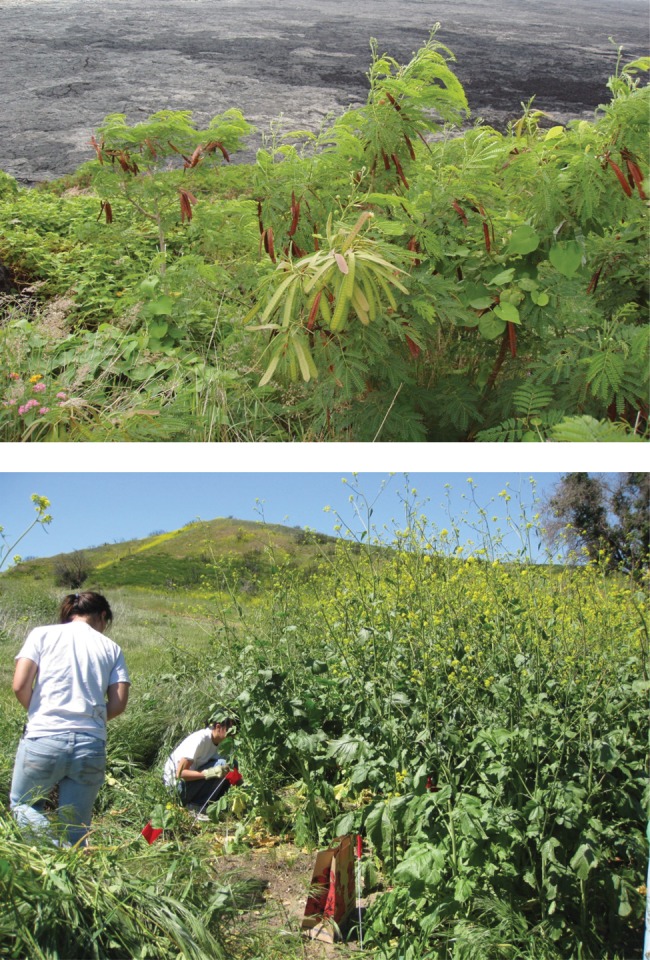
The legume *Leucaena leucocephala* invades young, low-nitrogen volcanic soils in Hawaii (top panel). Annual grasses and forbs, such as black mustard (*Brassica nigra*), aggressively invade semi-arid Mediterranean-climate ecosystems, such as southern California (bottom panel). Photo credit: Jennifer Funk.

Low-resource environments are defined as those where plant productivity is severely limited by light, water, or soil nutrient availability, such as forest understories, deserts, and ancient landscapes. In many of these ecosystems, native plants have evolved mechanisms to tolerate stress and to facilitate the extraction of limiting resources. These adaptations have resulted in a high degree of species richness and functional diversity in many low-resource ecosystems (Dallman, 1998; Lambers *et al.*, 2010; Olde Venterink, 2011). Native species appear to have a competitive advantage over invasive species in low-resource systems (Alpert *et al.*, 2000; Daehler, 2003), and communities become more susceptible to invasion when resource availability is increased (Davis *et al.*, 2000). While high-resource ecosystems tend to accumulate more exotic species than low-resource ecosystems (e.g. Huenneke *et al.*, 1990; Gross *et al.*, 2005; Stohlgren *et al.*, 2008), many invasive species do occur in low-resource ecosystems. For example, several legumes have successfully invaded low-nitrogen soils in Hawaii, and many annual grasses and forbs dominate semi-arid grassland and shrub systems in California (Fig. 1).

It is difficult to identify a suite of general traits explaining invasiveness, because traits of invaders depend on characteristics of the invaded habitats (Pysek *et al.*, 1995; Alpert *et al.*, 2000; Daehler, 2003; Pysek and Richardson, 2007; Tecco *et al.*, 2010). Specifically, the mechanisms allowing exotic species to invade low-resource ecosystems are likely to be very different from those allowing species to invade high-resource ecosystems. One way of thinking about invasion into low-resource environments is to focus on how plant species acquire and use resources. Competitive ability will be influenced by the ability of an individual to reduce the availability of a resource (e.g. resource acquisition, competitive effect, supply pre-emption), and by the ability to tolerate low resource availability (e.g. resource conservation, competitive response, concentration reduction; Tilman, 1982; Aarssen, 1983; Goldberg, 1990; Craine *et al.*, 2005). While there is much debate about which competition mechanism predominates across environments, research conducted over the last three decades suggests that plants in high-resource ecosystems succeed through high rates of resource acquisition, while species adapted to low-resource ecosystems largely display traits associated with resource conservation (Chapin, 1980; Craine, 2009). However, the dichotomy between resource acquisition and conservation is not clear in some low-resource ecosystems, as species can effectively acquire (e.g. specialized roots, high root density) and conserve resources (e.g. high tissue longevity, nutrient resorption; e.g. Sack *et al.*, 2003).

The trade-off between resource acquisition and conservation has been formalized in the leaf economics spectrum (LES), which shows that relationships exist among several key traits across a broad range of species and different climates (Reich *et al.*, 1997; Wright *et al.*, 2004). Plant species with low leaf mass per unit area (LMA), high rates of carbon assimilation, high leaf nitrogen (N) content, and short leaf lifespan occupy one end of the spectrum (fast return on investment), while plant species with high LMA, low rates of carbon assimilation, low leaf N content, and long leaf lifespans occupy the other (slow return on investment). With respect to invasion, several researchers have suggested that invasive species are positioned closer to the fast-return end of the LES (Leishman *et al.*, 2007; Penuelas *et al.*, 2010; Ordonez and Olff, 2012; but see Funk and Vitousek, 2007; Dawson *et al.*, 2011). This ‘fast-return’ strategy seems at odds with an ability to tolerate low-resource conditions, as species adapted to low-resource systems often display slow growth, resource-use efficiency, high LMA, high tissue construction cost, and long-lived tissues (Chapin, 1980; Vitousek, 1982; Coley *et al.*, 1985; Craine, 2009).

**Figure 2.**
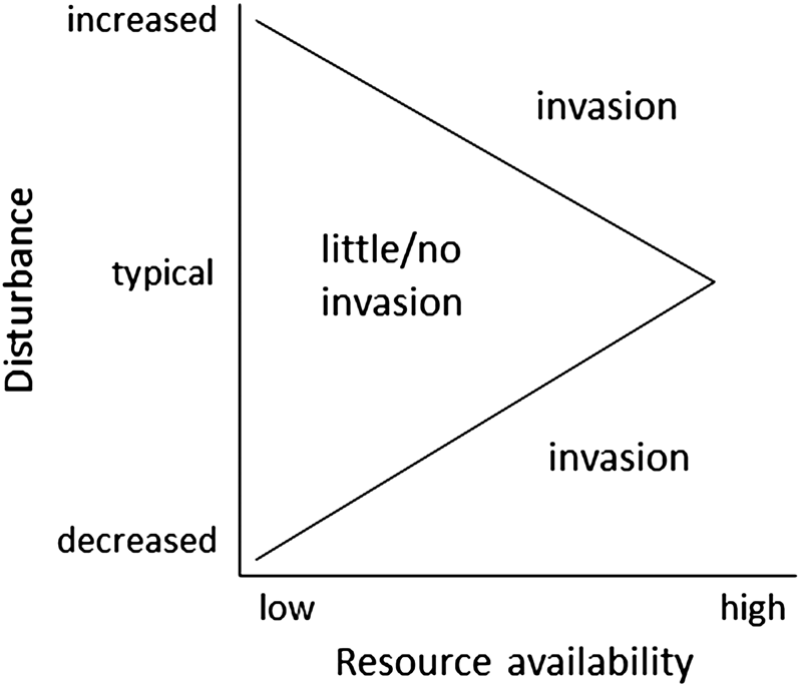
Model for interactive effects of resource availability and disturbance on habitat invasibility. Disturbance often increases resource availability by removing competitors. Decreased frequency of disturbance (e.g. fire suppression) can prevent succession from being reset and favour strongly competitive invasive species. Adapted from Alpert *et al.* (2000).

Do plant species invading low-resource ecosystems succeed through resource acquisition, resource conservation, or both? The theory of limiting similarity (MacArthur and Levins, 1967) predicts that invasive species will have different traits from native species and fill vacant niches (i.e. resource acquisition in the case of low-resource environments). In contrast, abiotic factors in low-resource environments are likely to constrain the range of possible traits (i.e. habitat filtering; Weiher *et al.*, 1998), resulting in invasive species with similar resource conservation traits to native species. Ultimately, the specific strategy or traits of successful invaders will depend on the type and frequency of resource limitation, disturbance regimes, propagule pressure, and a number of other factors (Sher and Hyatt, 1999; Alpert *et al.*, 2000; Theoharides and Dukes, 2007; Foxcroft *et al.*, 2011). Resource levels in many historically low-resource ecosystems around the world are increasing due to changing disturbance regimes. In many cases, disturbance increases resource availability, with potentially large impacts on invasibility (Alpert *et al.*, 2000; Fig. 2).

In this review, I summarize our understanding of resource acquisition and use in native and invasive species occurring in low-resource ecosystems. I focus on soil nutrients, water, and light as limiting resources. Lastly, I discuss how we can use our understanding of resource acquisition and use in native and invasive species to restore native plant communities.

## Soil nutrients

While plant growth can be limited by a number of macro- and micronutrients, the high mobility of N leads to N-limitation of plant growth in most ecosystems (Vitousek and Howarth, 1991). However, plant growth is often limited by phosphorus (P) availability in many tropical ecosystems with old, weathered soils. Additionally, plant species may be differentially limited by N and P in many systems. For example, plant growth in species with special adaptations for N (e.g. fixation) or P acquisition (e.g. cluster roots) may not be limited by the same nutrient as are neighbouring species (DiTommaso and Aarssen, 1989; Koerselman and Meuleman, 1996). Species also vary in their nutrient requirements. For example, grasses require lower amounts of P than forbs, possibly due to lower nucleic acid requirements associated with basal meristem leaf growth (Halsted and Lynch, 1996). Grasses with a C4 photosynthetic pathway can also operate at a lower N concentration due to higher photosynthetic nitrogen use efficiency (PNUE, i.e. carbon assimilation per unit of N; Sage and Pearcy, 1987).

The occurrence and degree of nutrient limitation in ecosystems is notoriously difficult to determine, because it depends on the process (e.g. plant growth) and time scale considered (Güsewell, 2004). Nutrient limitation is typically demonstrated when the addition of a nutrient increases plant growth (Vitousek and Howarth, 1991). As these types of experiments can be time consuming and labour intensive, element concentrations and ratios (e.g. N:P) of plant tissue have been used to demonstrate nutrient limitation in a variety of vegetation types. Across a diversity of ecosystems, N limitation is indicated by vegetation N:P ratios <10, P limitation is indicated by N:P ratios >20, and N and P can co-limit plant growth in between (Güsewell, 2004). Many researchers have also proposed specific N and P concentrations that characterize severely nutrient-limited soils. For example, N concentrations <13 mg g^−1^ and P concentrations <1 mg g^−1^ have been demonstrated to be limiting to plant growth (Wassen *et al.*, 1995; Güsewell and Koerselman, 2002).

Many species can invade low-nutrient soils, and the best-studied examples are in ecosystems with young volcanic soils (e.g. Vitousek and Walker, 1989; Mack *et al.*, 2001; Funk and Vitousek, 2007; Schoenfelder *et al.*, 2010), grasslands (e.g. Drenovsky *et al.*, 2012b; Han *et al.*, 2012), and arid shrublands (James and Richards, 2006). However, very few invaders can invade severely nutrient-deficient soils. For example, there are very few invaders (e.g. *Pinus*) in Australia where soil P levels are below 200 p.p.m. Plants require a high activity of RNA to sustain rapid rates of protein synthesis (growth-rate hypothesis; Elser *et al.*, 1996). Thus, invaders in P-limited systems should not be stereotypical fast-growing weeds. Historically, there have been fewer invasive species in saline- or serpentine-derived soils, which are characterized by low concentrations of macronutrients or high concentrations of salt or heavy metals (Lonsdale, 1999; Hoopes and Hall, 2002; Williamson and Harrison, 2002).

### Efficiency of nutrient use

**Figure 3.**
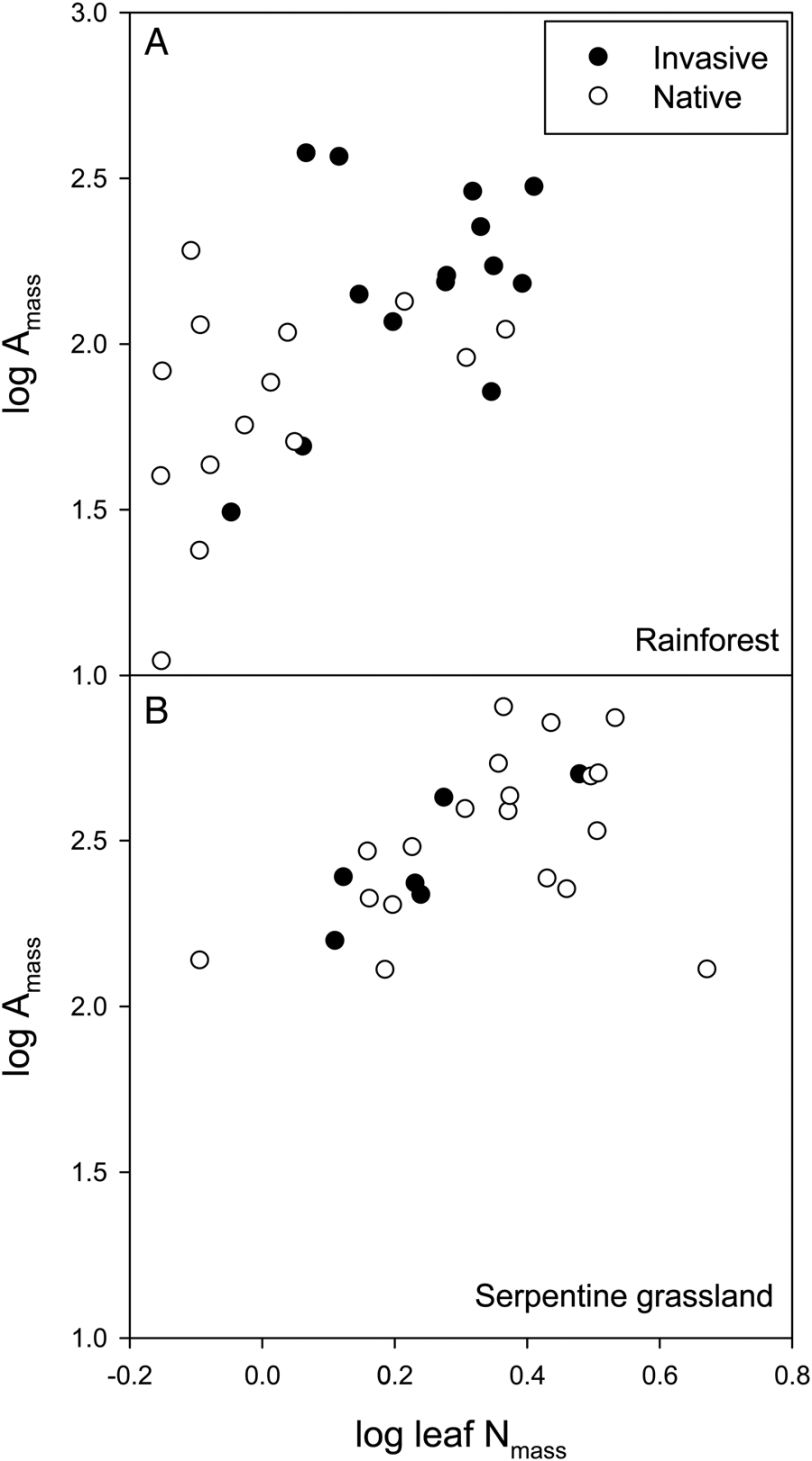
The relationship between mass-based photosynthetic rate (A_mass_) and leaf N content on a mass basis. Annual and perennial herbaceous and woody invasive species occupy the ‘high-return’ end of the spectrum in a rainforest in Hawaii (*r* = 0.59, *P* = 0.001; **A**); however, invasive grasses and forbs are similar to natives in a serpentine grassland in northern California (*r* = 0.47, *P* = 0.02; **B**). Data are from Funk and Vitousek (2007) and J. L. Funk (unpublished data).

Across species, there is a positive correlation between leaf N and photosynthetic rate (Fig. 3; Field and Mooney, 1986). Researchers working across low- and high-nutrient environments have found that native species occupy the lower left corner of this relationship (slow return), while invasive species occupy the upper right (fast return; e.g. Leishman *et al.*, 2007; Penuelas *et al.*, 2010; Ordonez and Olff, 2012). However, this pattern has not been demonstrated in all communities examined. For example, this generalization holds for species occurring in N-limited Hawaiian rainforest (Fig. 3A), but the pattern does not hold for annual grasses and forbs occurring in serpentine soils in California (Fig. 3B). If the slope of the relationship between carbon assimilation and leaf N is similar for both native and invasive species, then physiological processes are similar; more leaf N leads to a corresponding increase in photosynthesis. However, if the two groups display different slopes, this means that PNUE is higher for one group, which implies that native and invasive species have different biochemical or morphological traits.

The majority of studies examining nutrient-use efficiency in invasive species relative to co-occurring native species have found higher values in invasive species (Table 1). For example, Godoy *et al.* (2011) found higher PNUE in 20 Mediterranean invaders relative to natives in both low- and high-N conditions. Invasive lovegrass (*Eragrostis curvula*) had a higher PNUE relative to native grasses in low-nutrient soils in Australia (Firn *et al.*, 2012). Likewise, when grown in low-P conditions, invasive members of the genus *Pinus* had higher PNUE than non-invasive members (Matzek, 2011). However, a handful of studies have found no differences in PNUE between native and invasive species (Table 1). For example, Schoenfelder *et al.* (2010) found that an invasive forb (*Hypochaeris radicata*) growing on nutrient-poor volcanic soils did not have higher PNUE relative to a confamilial native species. Instead, the researchers proposed that *H. radicata* invades this low-N system through superior N acquisition and by diluting tissue N in order to build more photosynthetic structures.

**Table 1.** The number of studies that have observed trait differences between invasive and native or non-invasive exotic species in environments with (A) low soil nutrient availability, (B) low water availability and (C) low irradiance.

	Invasive > Native	No difference	Native > Invasive	References
**A. Low nutrient availability**				
*Resource conservation*				
High NUE	12	7	0	1-3,7-9,11,13,16,19, 20,27,28,31-33,42,44,49
High LMA	0	3	9	1-3,8,11,13,15,20,25,31,42,46
High LLS	0	1	1	15,35
High resorption	0	3	0	8,16,35
*Resource acquisition*				
High R:S	4	5	2	2,7,8,13,25,29,30,32,35,44,46
High uptake per mass	0	3	0	29,32,34
Mycorrhizae				not enough data, but see 43
Underutilized nutrient forms				not enough data
Specialized roots*				not enough data
**B. Low water availability**				
*Resource conservation*				
High WUE	1	5	2	4,5,8,16,17,33,48,49
High LMA	0	3	5	2,5,8,15,17,45,48,49
High LLS				not enough data, but see 15
Water storage				not enough data
Specialized leaf morphology**				not enough data
*Resource acquisition*				
High R:S	4	3	1	2,6,8,17,21,22,45,48
Early phenology	3	0	0	22,26,51
Mycorrhizae				not enough data, but see 43
Deep roots				not enough data
High SRL				not enough data
Fast tissue turnover				not enough data
**C. Low irradiance**				
*Resource conservation*				
High quantum yield	3	7	1	13,14,16,18-20,24,38,39,41,47
High LMA	1	5	11	9,13-15,18-20,24,27,36, 38,41,45-47,50,52
High LLS	3	0	2	9,12,15,23,27
High A/R_d_	4	3	1	9,20,24,27,33,36,38,41
*Resource acquisition*				
Low R:S	4	4	0	13,19,38,40,41,45,46,50
High chlorophyll content	2	2	1	9,14,18,39,40
Low CC/High PEUE	7	1	0	1,3,16,24,33,36,37,47

* Examples include nitrogen fixation and cluster roots; ** examples include low stomatal density, thick cuticle, trichomes. Abbreviations are: A/Rd, ratio of photosynthetic rate to dark respiration rate; CC, leaf construction cost; LMA, leaf mass per unit area; LLS, leaf lifespan; NUE, nutrient use efficiency; PEUE, photosynthetic energy use efficiency; R:S, root to shoot biomass ratio; SRL, specific root length; WUE, water use efficiency.

^1^Baruch and Goldstein 1999, ^2^Baruch and Jackson 2005, ^3^Baruch *et al.* 2000, ^4^Brock and Galen 2005, ^5^Cordell *et al.* 2002, ^6^DeFalco *et al.* 2003, ^7^Drenovsky *et al.* 2008, ^8^Drenovsky *et al.* 2012b, ^9^Durand and Goldstein 2001, ^10^Feng *et al.* 2007, ^11^Firn *et al.* 2012, ^12^Fridley 2012, ^13^Funk 2008, ^14^Funk and McDaniel 2010, ^15^Funk and Throop 2010, ^16^Funk and Vitousek 2007, ^17^Funk and Zachary 2010, ^18^Funk *et al.* 2013, ^19^Gleason and Ares 2004, ^20^Godoy *et al.* 2011, ^21^Grotkopp and Rejmanek 2007, ^22^Han *et al.* 2012, ^23^Harrington *et al.* 1989, ^24^Heberling and Fridley 2013, ^25^James and Drenovsky 2007, ^26^Kimball *et al.* 2011, ^27^Kloeppel and Abrams 1995, ^28^Laungani and Knops 2009, ^29^Leffler *et al.* 2011, ^30^Leishman and Thomson 2005, ^31^Leishman *et al.* 2010, ^32^Matzek 2011, ^33^McDowell 2002, ^34^Meisner *et al.* 2011, ^35^Morris *et al.* 2011, ^36^Nagel and Griffin 2004, ^37^Osunkoya *et al.* 2010a, ^38^Osunkoya *et al.* 2010b, ^39^Pammenter *et al.* 1986, ^40^Paquette *et al.* 2012, ^41^Pattison *et al.* 1998, ^42^Pavlik 1983, ^43^Pringle *et al.* 2009, ^44^Schoenfelder *et al.* 2010, ^45^Schumacher *et al.* 2008, ^46^Schumacher *et al.* 2009, ^47^Shen *et al.* 2011, ^48^Steers *et al.* 2011, ^49^Stratton and Goldstein 2001, ^50^van Kleunen *et al.* 2011, ^51^Wolkovich and Cleland 2011, ^52^Yamashita *et al.* 2000.

Few studies have examined the mechanisms of higher nutrient-use efficiency in invasive species. Plant species vary greatly in how they allocate N among photosynthetic and non-photosynthetic compounds in the leaf, and it is possible that invasive species with high PNUE allocate more N to photosynthetic compounds. Plants may allocate 5–32% of leaf N to ribulose-1,5-bisphosphate carboxylase oxygenase (photosynthetic) and 2–30% to cell walls (non-photosynthetic), with higher amounts of cell-wall protein occurring in longer-lived leaves (Evans, 1989; Warren and Adams, 2001; Onoda *et al.*, 2004; Takashima *et al.*, 2004; Harrison *et al.*, 2009). Feng *et al.* (2009) found that, compared with native populations, invasive populations of *Ageratina adenophora* allocated more N to soluble protein at the expense of cell-wall protein, which increased PNUE. A study of five native and five invasive woody species from Hawaii also found that invasive species allocate less N to cell-wall protein than native species (Funk *et al.*, 2013). While soluble protein content and PNUE did not differ between native and invasive species groups, invasive species allocated more N to amino acids, which may be used for rapid growth (Funk *et al.*, 2013).

Leaf longevity and nutrient recycling may influence nutrient-use efficiency on longer time scales. Invasive species in low-nutrient systems tend to have lower LMA, but this does not seem to translate into shorter leaf lifespan (Table 1). While there are very few data on nutrient recycling, nutrient resorption appears to be similar among native and invasive species (Table 1). Similar levels of N or P resorption have been found between native and invasive grass and forb species from the Intermountain West of the USA (Drenovsky *et al.*, 2012b), in invasive species of *Acacia* from Australia relative to co-occurring woody native species (reviewed by Morris *et al.*, 2011), and in a structurally and taxonomically diverse group of native and invasive species occurring in low-nutrient soils in Hawaii (Funk and Vitousek, 2007).

### Nutrient acquisition

Species occurring in N- and P-limited soils may possess morphological and physiological traits that facilitate N and P acquisition. Plants can maximize N uptake by increasing total root length, increasing specific root length, increasing root longevity, stimulating microbial decomposers via rhizodeposition, or allocating carbon to mycorrhizae. Few studies have surveyed root traits in native and invasive species and the existing data do not show clear differences between groups in root to shoot biomass ratio (R:S) or rates of nutrient uptake (Table 1). A high total root length appears to be more important in acquiring N than P (Olde Venterink and Güsewell, 2010). Instead, many native plants in P-limited soils have cluster roots and/or high phosphatase production in roots (Richardson *et al.*, 2009; Olde Venterink, 2011). It is unclear whether invasive species in P-limited systems share these strategies, although several *Lupinus* species have cluster roots and invade low-P soils in Australia (Lambers *et al.*, 2013).

Native species in P- and N-limited soils frequently form associations with mycorrhizal fungi, which help plants to sequester P and N and may also protect them from soil pathogens and drought stress (Auge, 2001; Willis *et al.*, 2013). A review of the limited data on mycorrhizal dynamics in native and invasive plant species suggested that many invasive plants do not associate with mycorrhizae, are facultatively mycorrhizal, or can partner with various types (arbuscular mycorrhizae vs. ectomycorrhizae) and species of fungi (Pringle *et al.*, 2009). Patterns appear to vary by region. An analysis of the California flora concluded that fewer invasive species than native species form mycorrhizal associations, while the pattern was reversed in Great Britain (Pringle *et al.*, 2009). There are examples of obligate mycorrhizal invasive species that use novel species of mycorrhizal fungi in the introduced habitat to outcompete native species (e.g. *Centaurea maculosa*; Marler *et al.*, 1999). Conversely, there are examples where novel mycorrhizal symbionts inhibit the growth of invasive species (e.g. *Plantago lanceolata*; Bever, 2002). There are also examples where an invasive species negatively affects neighbouring native species by disrupting mycorrhizal associations (e.g. Vogelsang and Bever, 2009; Meinhardt and Gehring, 2012). Understanding how native and invasive species associate with mycorrhizae is critical in nutrient-poor soils, and more data are needed to understand taxonomic and geographical patterns among species.

Many successful invaders in N-limited systems have symbiotic associations with N-fixing bacteria. For example, *Myrica faya*, *Leucaena leucocephala*, and other nitrogen-fixing species have invaded young, N-limited volcanic soils in Hawaii, filling an empty niche, because no native nitrogen-fixing species occur during primary succession on these soils (Fig. 1; Vitousek and Walker, 1989). Another example is Australian *Acacia* spp. that invade low-nutrient coastal dunes in Portugal (Rodríguez-Echeverría *et al.*, 2009) and low-nutrient fynbos in South Africa (e.g. Witkowski, 1991; Yelenik *et al.*, 2004). While nitrogen fixation may facilitate the invasion of these species into low-N ecosystems, nitrogen-fixing species may possess other traits that increase access to below-ground resources. For example, invasive Australian acacias allocate more biomass below ground (higher root mass ratio and root depth) compared with co-occurring native species, allowing them greater access to water and nutrients (Witkowski, 1991; Morris *et al.*, 2011). There is also evidence that some invasive species may nodulate more readily and fix greater amounts of N than co-occurring N-fixing species (Rodríguez-Echeverría *et al.*, 2009), although it is not known whether greater nodulation arises through a plant's ability to form associations with multiple bacterial partners (e.g. greater symbiotic promiscuity) and nodulate with low bacterial population sizes, or through differences in the bacteria themselves. Bacteria genera and strains vary in growth rate and the efficiency of N-fixation (e.g. Simms *et al.*, 2006). For example, invasive Australian acacias mainly associate with slow-growing *Bradyrhizobium*, but have been found occasionally to associate with fast-growing *Rhizobium* (Rodríguez-Echeverría *et al.*, 2011). Very little is known about how native and invasive species associate with different strains of N-fixing bacteria, and this is an interesting area for future research.

Lastly, invasive species may access forms of nutrients that neighbouring species are not using, including amino acids (Lipson and Näsholm, 2001). Very few studies have examined how native and invasive species compete for HN_4_^+^, NO_3_^−^, and organic N. Aanderud and Bledsoe (2009) concluded that invasive grass species used HN_4_^+^, the dominant form of N, forcing subordinate native species to use NO_3_^−^ and amino acids. Leffler *et al.* (2011) found that an invasive annual grass (*Bromus tectorum*) had a high mass-specific absorption rate and a high rate of whole-plant N uptake, implying that this species could access NO_3_^−^ that other species (including one exotic invasive and three native species) could not use. The potential for invasive species to use different forms of N is another exciting area for research, although organic N uptake may be restricted to cold, wet environments with low rates of N mineralization (Craine, 2009).

## Water availability

Arid environments (e.g. deserts, tundra, xeric shrubland) are characterized by < 250 mm of annual precipitation, while semi-arid environments (e.g. grassland, savanna, Mediterranean shrubland, seasonally dry tropical forests) receive 250–500 mm of annual precipitation (Holdridge, 1967). The degree to which water and N co-limit plant growth in arid systems has been investigated by several researchers (DeFalco *et al.*, 2003; James and Richards, 2005, 2006; Barker *et al.*, 2006), and results suggest that water availability most strongly limits plant growth in normal precipitation years, while N availability limits plant growth in wet years. Plants cope with water limitation by integrating biochemical, physiological, and morphological processes across multiple levels of organization (i.e. cell, organ, plant). As I discuss below, there is evidence that some invasive species possess drought-tolerant traits, while others do not.

### Efficiency of water use

Many leaf-level traits, including thick cuticles and trichomes, function to reduce the amount of water lost from leaves (Sandquist and Ehleringer, 1998). Additionally, plants in arid environments tend to have high LMA. Across taxonomically diverse plant species, high LMA leads to lower leaf-level carbon assimilation rates, representing one of the key trade-offs of the LES (Reich *et al.*, 1997; Wright *et al.*, 2004). However, high LMA in arid systems has been linked to larger amounts of mesophyll tissue (which contains the photosynthetic machinery) rather than higher amounts of structural tissue (Wright and Westoby, 2002). Thus, plants can increase their water-use efficiency (WUE) by investing more resources in photosynthetic enzymes and pigments to draw down intercellular CO_2_ concentrations and reduce transpiration loss (Westoby *et al.*, 2002). Given that photosynthetic enzymes require N, plants adapted to arid regions generally have high leaf N content, presumably to increase WUE (Wright and Westoby, 2002). Thus, LMA and photosynthetic rate are not necessarily negatively correlated in arid and semi-arid systems (e.g. Steers *et al.*, 2011).

The existing data suggest that LMA is lower in invasive species than in native species occurring in arid environments (Table 1). For example, in the Mojave desert, two annual exotic grasses (*Bromus madritensis* and *Schismus barbatus*) and one annual exotic forb (*Erodium cicutarium*) produce many thin (low LMA) leaves (Steers *et al.*, 2011). Thin leaves generally have low quantities of structural carbohydrates, which results in low energetic or construction cost of the leaf (Griffin, 1994). Low construction cost is often associated with higher plant growth rates (Nagel and Griffin, 2004), because resources are available to produce more photosynthetic tissue, which maximizes plant-level carbon assimilation. However, while cheap structures may provide an initial growth advantage, more leaf area leads to higher plant-level transpiration rates, and this may render these exotic species more prone to water stress in low-precipitation years. Nevertheless, many annual species (particularly invasive annuals) may employ this strategy, where the production of cheap structures facilitates a rapid response to unpredictable precipitation events (Angert *et al.*, 2007; Huxman *et al.*, 2008). This mechanism may explain why exotic species can spread in wet years and remain in the seed bank during dry years (e.g. *Pennisetum setaceum* in Hawaii; Cordell *et al.*, 2002).

Cavaleri and Sack (2010) conducted a meta-analysis of 40 studies examining water use in native and invasive plants worldwide and found similar values for leaf-level WUE expressed on an instantaneous basis (photosynthetic rate/transpiration rate) and integrated over leaf lifespan (δ^13^C). This same pattern emerges when the analysis is restricted to arid and semi-arid systems (Table 1). Cavaleri and Sack (2010) also found that invasive species had lower pre-dawn water potential (ψ_pd_) than native species, particularly in regions with low mean annual precipitation. They suggest that invasive species may favour drier microsites within habitats, deplete soil moisture levels more than natives, or have higher nocturnal transpiration (e.g. Dawson, 1993).

### Water acquisition and drought tolerance

Root depth, root to shoot biomass ratio (R:S), and mycorrhizal associations strongly influence water uptake. The influence of rooting depth on plant performance depends on the magnitude and frequency of precipitation; more frequent large precipitation events increase the productivity of deep-rooted shrubs, while more frequent small events increase the productivity of shallow-rooted species (Weltzin and McPherson, 1997; Gebauer and Ehleringer, 2000; Loik, 2007). I am not aware of any study that has quantified root depth for native and invasive species in an arid system. Several studies have demonstrated enhanced water acquisition in species invading arid and semi-arid systems through higher R:S (Table 1). For example, a study of 12 phylogenetically controlled pairs of native and invasive woody species in California found that invasive species allocated more biomass to roots, which may help them tolerate summer drought (Grotkopp and Rejmanek, 2007). Additionally, in a study of annual species from the Mojave Desert, DeFalco *et al.* (2003) found that *Bromus madritensis* uses more water, takes up water at a faster rate, and draws down soil water content to a lower level than neighbouring native forb and grass species due to greater biomass allocation below ground and greater root surface area. These same traits also confer an advantage in N acquisition; *B. madritensis* had a higher N content and N uptake rates in some treatment conditions (DeFalco *et al.*, 2003). Given that species adapted to arid and semi-arid environments must maintain a high N status to achieve high WUE (Wright and Westoby, 2002), the ability to take up N during precipitation events may strongly impact plant N status and, consequently, plant fitness.

Understanding species responses to short-term changes in water availability is important because global climate models project intensified intra-annual variation in precipitation in many arid environments, resulting in larger precipitation events with longer intervening dry periods (Diffenbaugh *et al.*, 2005; Knapp *et al.*, 2008). Han *et al.* (2012) found a higher R:S in invasive species relative to native species in variable irrigation conditions, suggesting that invasive species may demonstrate enhanced physiological plasticity to changing environments. In contrast, a study of native and invasive shrubs in southern California found no clear differences in how water-stressed individuals of these groups responded to precipitation events (Funk and Zachary, 2010). One native (*Salvia mellifera*) and one invasive species (*Ricinus communis*) displayed rapid photosynthetic recovery following drought, but this was attributable to enhanced leaf-level function (WUE) rather than new root growth.

Much of the work on water acquisition in native and invasive species has focused on root traits rather than differences in water conductance through the xylem. Across taxonomic groups, there appears to be a trade-off between water conductance and vulnerability to cavitation (Hacke *et al.*, 2001; Preston and Ackerly, 2003). While reinforcement of water-conducting vessels and tracheids prevents xylem cavitation at low water potential, transport efficiency is reduced by increased wall thickness in reinforced cells. Woody species adapted to arid and semi-arid environments may reduce water conduction in order to prevent cavitation, and it is unknown whether woody species invading these environments are similar to native species in this way. Caplan and Yeakley (2010) found that an invasive blackberry (*Rubus armeniacus*) maintained higher stomatal conductance and lower hydraulic resistance throughout the year relative to two native congeners. Greater rates of water transport were probably driven by access to deeper water sources and shoot water storage, although species differences in stomata anatomy and xylem embolism were not examined.

### Phenology

Plants growing in arid and semi-arid systems display a broad range of phenological patterns that limit the severity of water stress (Williams *et al.*, 1997; Sandquist and Ehleringer, 1998). Annual species are common in arid systems, because this strategy enables them to complete a short life-cycle during the favourable wet season. Many perennial species are drought deciduous, which allows them to be dormant during the hot, dry summer months (Nilsen and Muller, 1981; Comstock and Ehleringer, 1986). However, drought deciduousness is more economically feasible in nutrient-rich environments, where the costly loss of nutrients in shed leaves does not adversely affect plant fitness (reviewed by Morris *et al.*, 2011).

Plant species may cope with fluctuations in the timing and magnitude of water availability by altering their phenology. An analysis of several US plant databases found that exotic species generally develop leaves earlier in the year than natives, which may allow them to pre-empt resources by being active earlier (Table 1; Wolkovich and Cleland, 2011). For example, African lovegrass (*Eragrostis curvula*), which occupies nutrient- and water-depleted sites in Australia, germinates and grows faster than functionally similar native grass species (Han *et al.*, 2012), potentially enhancing its competitive ability. Phenological patterns also correlate with plant function. In the Sonoran Desert, Kimball *et al.* (2011) found that annual species with high WUE, including the invasive forb *Erodium cicutarium*, germinate earlier in the growing season and reproduce for a longer time period. Species with low WUE germinate later, following several rainfall events, but the plants experience higher risk of mortality associated with warmer temperatures later in the growing season.

With respect to phenology, evergreen species may be more constrained in their ability to respond morphologically to precipitation events than deciduous perennials or annual species (Grime *et al.*, 1986). However, retaining long-lived leaves may provide evergreen species with an advantage over those that must produce new leaves following a precipitation event. For example, *Eragrostis lehmanniana*, an invasive grass in the southwest USA, can up-regulate photosynthesis quickly following summer precipitation events, while a co-occurring native bunchgrass, *Heteropogon contortus*, lags behind as it grows new leaves (Ignace *et al.*, 2007). In contrast, species with short-lived or inexpensive tissues can track the limiting resource over time and invest in tissues that are more appropriate for the new environment. While this has primarily been demonstrated for variation in light availability (Ackerly and Bazzaz, 1995), there is some evidence of a stress-tracking ability in response to drought as well. Two exotic species (*Ricinus communis* and *Nicotiana glauca*) in a semi-arid coastal sage scrub community in southern California responded to drought-induced high-light stress with new growth and large decreases in the function of existing leaves (photosynthetic rate, light harvesting), suggesting that these species respond to stress by turnover of existing tissue rather than acclimatization of existing tissue (Funk and Zachary, 2010).

## Light availability

Plant species occurring in low-light environments demonstrate a trade-off between shade tolerance and growth rate (Bazzaz, 1979; Valladares and Niinemets, 2008). Shade-intolerant species grow rapidly in order to reach higher light levels at the top of the canopy and display high photosynthetic rates, early reproduction, and short lifespan. Some shade-intolerant exotic species can take advantage of disturbances that create high-light gaps. The exotic tree species *Ailanthus altissima* is shade intolerant (Martin *et al.*, 2010) but succeeds in low-light forests because it requires only a small gap to initiate rapid growth to reach the canopy (Knapp and Canham, 2000). Higher leaf area ratios and low R:S, characteristics of many species invading forests (Table 1; Pattison *et al.*, 1998; Standish *et al.*, 2001; Reinhart *et al.*, 2006; Schumacher *et al.*, 2009; Paquette *et al.*, 2012), suggest that these species are able to take advantage of high-light conditions and grow rapidly in response to natural or human-induced canopy gaps. For example, Leicht and Silander (2006) found that an invasive liana (*Celastrus orbiculatus*) grew taller than a congeneric native (*Celastrus scandens*), which allows it to forage more efficiently for canopy gaps. Once established, some shade-intolerant species change the structure of the forest, promoting high-light conditions that favour exotic species. For example, understory species in forests can create an environment suitable for them by suppressing recruitment of native canopy species (Standish *et al.*, 2001; Funk and McDaniel, 2010). However, most species invading forests are shade tolerant (Martin *et al.*, 2009), and therefore shade tolerance is the focus of the following section.

### Shade tolerance

Researchers have characterized the traits associated with shade tolerance, although the focus has been on leaf traits as opposed to shoot and root traits (Mooney, 1972; Bjorkman, 1981; Chazdon, 1988; Givnish, 1988). Species adapted to low-light environments possess a suite of physiological traits to maximize light capture, such as high quantum yield (carbon assimilated per photon absorbed), high chlorophyll content, low respiratory rates, low light compensation points, and allocation of nitrogen to proteins associated with light-harvesting functions at the expense of carbon-assimilation functions (Bjorkman, 1981; Evans and Poorter, 2001; Givnish *et al.*, 2004; Craine, 2009; but see Walters and Reich, 2000; Janse-ten Klooster *et al.*, 2007; Wyka *et al.*, 2007). Shade-tolerant species typically possess leaves with large amounts of structural tissue, which helps protect them against physical stresses and herbivory (Lusk and Warton, 2007). This increases LMA, leaf longevity and the lifetime carbon assimilation of the leaf (Reich *et al.*, 1997; Westoby *et al.*, 2002), which is advantageous in low-light habitats on long time scales.

There have been few publications on understory invaders despite their presence, and they may have been overlooked historically because they do not spread quickly or possess traits more commonly ascribed to fast-growing exotic species (Martin *et al.*, 2009). While some studies in low-light environments have found higher photosynthetic rate and quantum yield in invasive species relative to native species, the majority of studies found no significant differences in these traits (Table 1). Some invasive species may achieve high rates of carbon assimilation at low irradiance by allocating more resources to the light-harvesting components of photosynthesis, such as chlorophyll (Table 1; Durand and Goldstein, 2001). However, increasing photosynthetic capacity can be costly. High metabolic activity leads to higher respiratory costs resulting from higher rates of protein turnover and maintenance of solute gradients required for phloem loading (Lambers *et al.*, 2008); thus, leaves with high photosynthetic rates typically have higher light compensation points associated with higher respiratory costs (e.g. Givnish *et al.*, 2004). However, several studies of photosynthetic function in native and invasive species found that invasive species in low-light conditions achieved high photosynthetic rates at a low respiratory cost (Table 1). As the mechanism for this pattern was not examined in these studies, there is a need for additional research in this area.

Invasive species in low-light environments have lower LMA and leaf construction costs relative to native species (Table 1). When coupled with higher photosynthetic rates, lower construction costs can increase photosynthetic energy-use efficiency (PEUE; carbon assimilated per unit of energy invested in leaf construction). Building cheaper leaves allows a plant to produce more photosynthetic structures for the same energy cost, which maximizes whole-plant carbon gain. However, there is a trade-off between low construction cost and leaf lifespan, in that cheaper leaves often have lower leaf lifespan. Long leaf lifespan is a characteristic of shade-tolerant species, because this allows a plant to assimilate carbon over a longer time period for the same initial investment in leaf construction. Several surveys of native and exotic species in forests have found earlier bud break and longer leaf lifespans in exotic species (Harrington *et al.*, 1989; Kloeppel and Abrams, 1995; Fridley, 2012; but see Durand and Goldstein, 2001; Funk and Throop, 2010), which seems at odds with the general pattern of lower leaf construction cost in invasive species. Nevertheless, the effect on PEUE is the same, in that longer leaf lifespan will increase PEUE as more carbon is assimilated per resource invested over time.

Measures of resource-use efficiency integrated over a leaf's lifespan suggest very different scenarios for the success of invasive species compared with the instantaneous measures (PNUE, WUE, and quantum yield) that I have presented thus far. Instantaneous measures of resource-use efficiency may reflect performance on short time scales, while measures integrated over leaf lifespan may more accurately reflect performance on longer time scales (e.g. multiple seasons; Funk and Vitousek, 2007). The appropriate measure of resource-use efficiency will be context dependent, and this may partly explain discrepancies across studies, such as the finding that some invasive species in low-light environments display lower leaf construction costs, while others maximize leaf lifespan.

### Shade tolerance and rapid growth: the best of both worlds

Several studies have found that invasive species do not adhere to the growth rate–shade tolerance trade-off (see Table 2 of Catford *et al.*, 2012). Norway maple (*Acer platanoides*), one of the most common forest invaders in the northeastern USA and in riparian and montane forests in the northern Rocky Mountains in the USA, is a well-studied species that displays both high survivorship in low-light conditions (2% full sun) and high growth rates in high-light conditions (80% full sun; Martin *et al.*, 2010). The departure of *A. platanoides* from the growth rate–shade tolerance trade-off probably results from a combination of plant- and leaf-level traits. *Acer platanoides* has a lower R:S than co-occurring native species in high-light conditions (Paquette *et al.*, 2012) and in deeply shaded forests (Reinhart *et al.*, 2006). Allocation of biomass to photosynthesizing tissues can result in higher plant-level assimilation and growth, which is advantageous when plants are competing primarily for light. At the leaf level, *A. platanoides* has high rates of photosynthesis and high LMA compared with the native congener *Acer saccharum*, but the denser leaves result from more chloroplasts in the palisade and mesophyll cells rather than increased structural tissue (reviewed by Kloeppel and Abrams, 1995). Despite the increased allocation to photosynthetic tissue, the two *Acer* species did not differ in respiratory costs (Kloeppel and Abrams, 1995).

Other invasive species display both shade-tolerance traits and rapid growth. Durand and Goldstein (2001) found that an invasive tree fern (*Sphaeropteris cooperi*) had higher chlorophyll content (shade tolerance) and larger annual height growth compared with native tree ferns in Hawaii. Pammenter *et al.* (1986) compared an invasive and native *Agrostis* species on a light-limited sub-Antarctic island and found that the invasive species had higher light-use efficiency over a wide temperature range. Additionally, the invasive *Agrostis* had thinner leaves and allocated relatively more carbon to photosynthetic tissue, presumably resulting in higher plant-level assimilation and growth. As discussed above, this strategy is shared by several exotic annual species in arid environments. Lastly, when compared with native species of varying successional status, Yamashita *et al.* (2000) found that an exotic tree species (*Bischofia javanica*) responded faster physiologically (increased photosynthesis of existing leaves) and morphologically (new leaf formation) to increased light levels simulating a canopy gap. Furthermore, following a transition from shade to sun, *B. javanica* decreased leaf chlorophyll content and increased PNUE, suggesting that this species reallocates N from light-harvesting to carboxylation components of photosynthesis.

## Implications for restoration and conservation

Given that many low-resource environments have high species and functional diversity, it is essential to understand invasion dynamics in these systems in order to conserve and restore native biodiversity. While invasive species outperform native species in many communities, native species generally have an advantage or hold their own in low-resource environments (Daehler, 2003), which means that opportunities exist for control and restoration. Restoration techniques are diverse and range from methods that target specific invaders to those that manipulate community-level processes, such as disturbance, seed dispersal, and resource availability.

**Figure 4.**
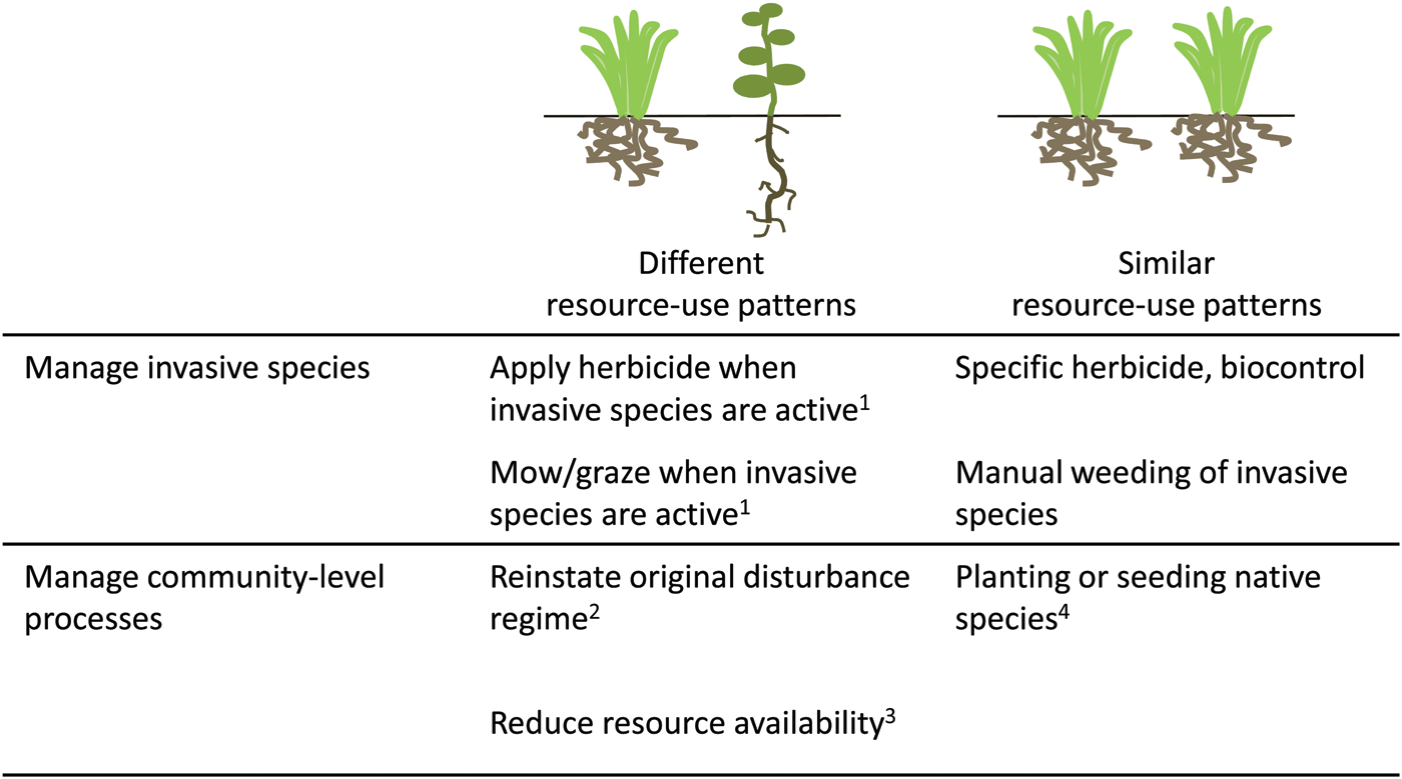
Traits associated with resource acquisition and use may suggest restoration strategies for invaded plant communities. Restoration approaches are separated into two categories, namely those that directly target invasive species and those that seek to alter a community-level process. ^1^When native and invasive species differ in the timing of germination or reproduction, practitioners can apply herbicide, mow, or graze during periods when invasive species are active or flowering. ^2^Original disturbance regimes should be restored when altered disturbance facilitates invasion, such as where canopy gaps increase light availability or fire reduces competition. ^3^Resource availability should be reduced when invasive species have higher resource requirements than native species. Examples include lowering soil nutrient availability by adding carbon to the soil, establishing canopy trees to reduce light, and tarping to reduce vertical or horizontal water flow. ^4^If native and invasive species are using resources in similar ways, but populations of native species are dispersal limited, practitioners can introduce native plants or seeds to overcome this barrier.

When native and invasive species differ in the timing or magnitude of resource acquisition or use, reinstating natural disturbance regimes or lowering resource availability may facilitate the restoration of native plant species (Fig. 4). For example, many studies have shown that adding carbon to soil can lower plant-available N and, consequently, reduce the abundance of invasive species (e.g. Blumenthal *et al.*, 2003; Corbin and D'Antonio, 2004; Cherwin *et al.*, 2009; Steers *et al.*, 2011; but see James *et al.*, 2011). Additionally, eliminating disturbance that creates canopy gaps in forests can exclude shade-intolerant invasive species (Funk and McDaniel, 2010; Emer and Fonseca, 2011). Community-level manipulations may be particularly effective if native and invasive species differ in the timing of resource use. Marushia *et al.* (2010) found that early season application of herbicide reduced exotic cover without affecting cover of native desert annuals. This method was effective because, as discussed above, many exotic species in desert systems display a rapid phenology and germinate before native species (Wolkovich and Cleland, 2011).

Community-level restoration approaches will be most effective when native and invasive species differ in the timing and magnitude of resource use (Emer and Fonseca, 2011; Steers *et al.*, 2011). As highlighted above, many invasive species have similar or higher resource-use efficiency compared with neighbouring native species. In these cases, lowering resource availability will not suppress the growth of invasive species. When confronted with resource-use-efficient invasive species, the best restoration options may be manual control of invasive species, planting or reseeding functionally similar native species, controlled burns, or herbicide treatment (Funk *et al.*, 2008; Fig. 4). A better understanding of physiological and morphological traits can help in the identification of possible restoration strategies in a given community (Funk *et al.*, 2008; Drenovsky *et al.*, 2012a).

## Conclusions

Invasion is a community-level process, and the traits of invasive species depend on many factors, including the traits of native species, as well as propagule pressure, and the type and frequency of disturbance and resource limitation. While there is significant variation in results from studies of invasive species conducted in low-resource systems, it is possible to make a few generalizations. With respect to resource conservation, invasive species appear to use nutrients more efficiently than natives in low-nutrient soils. However, invasive and native species are similarly efficient at using water and light in arid and light-limited systems, respectively. With respect to resource acquisition, invasive species tend to have higher R:S in arid systems and lower R:S in light-limited systems, relative to co-occurring native species. Additionally, invasive species have lower leaf construction costs and higher PEUE in light-limited systems. Earlier phenology in arid systems may also help invasive species to outcompete native species for resources.

There are several gaps in our understanding of how species invade low-resource systems. In low-nutrient systems, we need more information on how native and invasive species associate with different strains/species of N-fixing bacteria and mycorrhizae. Additionally, we have a limited understanding of how native and invasive species differ in the timing and form of nutrient use, as well as the capacity for recycling nutrients. Much of the research in arid and semi-arid ecosystems has focused on morphological traits (such as biomass allocation and LMA) and a few physiological traits (e.g. WUE), and we know very little about water relations in native and invasive species in these environments. The few studies that have examined physiology in species invading forest systems have focused on a few species (e.g. *A. platanoides*, *A. altissima*). More studies are needed to determine whether invasive species adhere to the shade tolerance–growth rate trade-off and to determine the physiological mechanisms underlying any deviations. For example, studies at the cellular level are needed to understand how some invasive species can increase carbon gain without a corresponding increase in respiratory costs.

Propagule pressure, trait plasticity, and the type of species comparison confound our understanding of invasion in low-resource systems. Firstly, invasion in low-resource ecosystems may be influenced by seed and vegetative dispersal. For example, in low-N fields, annual grasses can dominate, even though they are weak competitors relative to native perennial grasses, because native species are dispersal limited (Seabloom *et al.*, 2003). Secondly, plant species may benefit from physiological or morphological plasticity in low-resource environments, where resources can vary temporally or spatially (e.g. Poorter and Lambers, 1986; Davis *et al.*, 2000; Valladares *et al.*, 2000; Balaguer *et al.*, 2001; Valladares *et al.*, 2002; Funk, 2008). Several studies suggest that invasive species can be more plastic than native species in specific environmental conditions (for review see Davidson *et al.*, 2011; Palacio-López and Gianoli, 2011). Thus, caution should be used when interpreting trait data across environmental gradients, because species may differ in the plasticity of traits and, importantly, trait plasticity may not necessarily result in increased fitness or translate into increased abundance (Funk, 2008; Osunkoya *et al.*, 2010b; Godoy *et al.*, 2011; van Kleunen *et al.*, 2011; Dawson *et al.*, 2012; Firn *et al.*, 2012; Matzek, 2012). Lastly, most of the studies that examined invasive species in low-resource environments have compared invasive species with co-occurring native species (but see Leishman and Thomson, 2005; Feng *et al.*, 2007; Matzek, 2011; Shen *et al.*, 2011; van Kleunen *et al.*, 2011). Comparisons of invasive and non-invasive exotic species answer different questions from conventional comparisons of native and invasive species (e.g. why some exotic species become invasive, while others do not; van Kleunen *et al.*, 2010), and our understanding of invasion in low-resource ecosystems could benefit from these types of comparisons.

Many low-resource environments are experiencing radical changes as N deposition and land-use legacies increase nutrient availability in low-N and low-P systems, climate change alters the frequency and magnitude of precipitation in arid and semi-arid systems, and deforestation alters light availability. The effects of global change factors and their interactions on invasive species are still largely unresolved, and more research is needed on this important topic. Understanding the physiological mechanisms by which native and invasive species respond to current and future resource availability will help restoration efforts. Specifically, leaf- and plant-level traits can suggest ways to manipulate community-level properties to restore invaded ecosystems.
